# Effect of Systemic Inflammation on Rat Attentional Function and Neuroinflammation: Possible Protective Role for Food Restriction

**DOI:** 10.3389/fnagi.2019.00296

**Published:** 2019-10-25

**Authors:** Brittney Yegla, Thomas Foster

**Affiliations:** ^1^Department of Neuroscience, McKnight Brain Institute, University of Florida, Gainesville, FL, United States; ^2^Genetics and Genomics Program, University of Florida, Gainesville, FL, United States

**Keywords:** aging, attention, caloric restriction, lipopolysaccharide, systemic inflammation

## Abstract

**Background**: Aging is characterized by subtle cognitive decline, which correlates with increased peripheral inflammation. Acute activation of the peripheral immune system, *via* lipopolysaccharide (LPS) injection, elicits deficits in hippocampal-dependent spatial memory. Little is known concerning the effect of chronic inflammation on prefrontal cortex (PFC)-dependent vigilance. We examined the impact of repeated LPS injections in young and middle-age rats on the 5-choice serial reaction time task (5-CSRTT), expecting repeated LPS treatment to induce attentional deficits with greater disruption in middle-age.

**Methods**: Male Fischer-344 rats, 4- and 12-months-old, were food restricted and trained on the 5-CSRTT. Once rats reached criterion, they were injected with LPS (1 mg/kg, i.p.) weekly for 4 weeks and testing started 48 h after each injection. To examine the possibility that mild food restriction inherent to the behavioral task influenced inflammation markers, a second group of food-restricted or *ad-lib*-fed rats was assessed for cytokine changes 48 h after one injection.

**Results**: Performing LPS-treated rats exhibited a sickness response, manifesting as reduced initiated and completed trials during the first week but recovered by the second week of testing. After the first week, LPS-treated rats continued to exhibit longer response latencies, despite no change in food retrieval latency, suggestive of LPS-induced cognitive slowing. Similarly, LPS-induced impairment of attention was observed as increased omissions with heightened cognitive demand and increased age. Repeated LPS-treatment increased the level of PFC IL-1α, and PFC IL-6 was marginally higher in middle-age rats. No effect of age or treatment was observed for plasma cytokines in performing rats. Histological examination of microglia indicated increased colocalization of Iba1+ and CD68+ cells from middle-age relative to young rats. Examination of food restriction demonstrated an attenuation of age- and LPS-related increases in plasma cytokine levels.

**Conclusions**: Systemic inflammation, induced through LPS treatment, impaired attentional function, which was independent of sickness and exacerbated by increased cognitive demand and increased age. Additional studies revealed that food restriction, associated with the task, attenuated markers of neuroinflammation and plasma cytokines. The results emphasize the need for improved methods for modeling low-level chronic systemic inflammation to effectively examine its impact on attention during aging.

## Introduction

Aging is characterized by a decline in hippocampal and prefrontal cortical (PFC) functions, resulting in impaired learning, memory, and executive function (Buckner, 2004; Disterhoft and Oh, 2006; Lister and Barnes, 2009; Harada et al., 2013; Guidi et al., 2015a; Kirova et al., 2015). Increased individual variability of cognitive function in aging suggests the involvement of genetic and lifestyle factors that mediate resiliency and the response to stress. For instance, regular exercise and caloric restriction have been shown to exhibit neuroprotective effects in aging (Mattson, 2012; Murphy et al., 2014). A consistent finding with advancing age is an increase in circulating inflammatory markers, which are negatively correlated with cognitive function (Weaver et al., 2002; Marsland et al., 2006; Rafnsson et al., 2007; Gimeno et al., 2008; Bettcher et al., 2012; Singh-Manoux et al., 2014; Lin et al., 2018). Although the mechanism by which systemic inflammation communicates with the brain is debated (Banks and Erickson, 2010; Kempuraj et al., 2017; Li et al., 2018; Zhang et al., 2018), an elevation in neuroinflammation is noted with aging, and acute infections (Chen et al., 2008; Henry et al., 2009; Kohman et al., 2010; Barrett et al., 2015; D’Avila et al., 2018) and cytokines have been shown to cross the blood brain barrier (reviewed in Banks and Erickson, 2010). This neuroinflammation manifests as reactive astrocytes and microglial cells, which further stimulate inflammatory cytokine signaling (Hoogland et al., 2015; von Bernhardi et al., 2015).

By utilizing lipopolysaccharide (LPS), the outer membrane of *E. coli*, to induce systemic inflammation, animal studies have demonstrated a direct negative impact of peripheral inflammation on hippocampal-dependent tasks of contextual memory, spatial memory, and object recognition, and older animals appear to be more sensitive to its disruptive effects (Tateda et al., 1996; Godbout et al., 2005; Kohman et al., 2007, 2010; Chen et al., 2008; Barrientos et al., 2009b; Tarr et al., 2011; Kahn et al., 2012; Czerniawski and Guzowski, 2014; Czerniawski et al., 2015; Ming et al., 2015; Solomon and Taghogho, 2017; Shen et al., 2018; D’Avila et al., 2018). However, some of these impairments are confounded by sickness behavior, manifesting as impaired motor function and thus impacts locomotor measures critical to interpreting cognitive changes. Chronic LPS treatment, on the other hand, results in the development of tolerance to sickness behavior while maintaining cognitive deficits (Kahn et al., 2012; Zhu et al., 2014).

In contrast to hippocampal-dependent cognition, there is little research on the effects of systemic inflammation on cognitive processes that depend upon the PFC, which displays enhanced susceptibility to disruption in aging (Culley et al., 2014; Shen et al., 2018). One such function, sustained attention, declines in aging and has recently been found to be impaired in relation to increased peripheral inflammatory markers across the lifespan (Beydoun et al., 2018). Moreover, studies that have examined the effects of peripheral inflammation are generally limited to acute effects of a single injection or “chronic” effects produced from a week of daily injections, both of which fail to model the repeated inflammatory activation and extended period of neuroinflammation observed in aging. Thus, the purpose of this study was to investigate the impact of repeated peripheral inflammation, *via* multiple LPS injections, on attentional function and its interaction with aging in a rat model. Due to the heightened inflammatory markers already present in aged rats, middle-age rats were selected for this study. We expected that repeated LPS treatment would induce attentional deficits, with greater disruption in middle age. In addition, due to the mild food restriction involved in the attentional task, rats were also evaluated for potentially mitigating effects of food restriction on inflammatory activation, though we predicted minimal effects with the low percentage of food restriction employed.

## Materials and Methods

### Subjects

**Figure 1:**
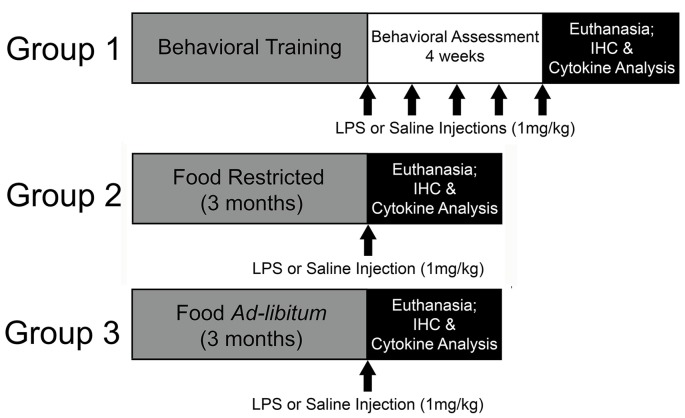
Experimental design. In this experiment, the impact of repeated systemic inflammation on attentional function was evaluated with Group 1, a cohort of young and middle age Fischer-344 rats that were food restricted and underwent behavioral training and testing on the 5-choice serial reaction time task (5-CSRTT). Once they reached criterion on the task they received injections of saline or lipopolysaccharide (LPS; 1 mg/kg, i.p.) once a week for 4 weeks while their attention was evaluated. They were injected once more 48 h prior to euthanasia. To examine the potential modifying effects of food restriction on inflammatory responsivity, two cohorts (Group 2 and 3) were either food restricted or maintained with full access to food for 3 months, which aligns with the average length of time for 5-CSRTT behavioral training. They were then injected with saline or LPS 48 h prior to euthanasia and tissue was collected for cytokine analysis and immunohistochemistry (IHC).

Young (4-months-old; *N* = 33) and middle-age (12-months-old; *N* = 42) Fischer-344 male rats were acquired from the National Institute of Aging (Bethesda, MD, USA) and housed at University of Florida’s animal facility on a 12:12 light/dark cycle (lights on: 7 AM) in temperature- and humidity-controlled rooms. Throughout the experiment, rats remained group-housed with free access to water and standard rat chow. Following a week of acclimation to the facility, two cohorts of rats were food restricted to 85% of their starting body weight. One cohort underwent behavioral training on the 5-choice serial reaction time task (5-CSRTT) to evaluate the impact of repeated peripheral inflammation on attentional function (young: *N* = 14; middle-age: *N* = 22). To examine the effect of acute peripheral inflammation (i.e., single injection) and caloric restriction as a potential mediator of peripheral and central inflammatory responses, a second and third cohort of young and middle-age rats were included, one of which was food restricted for 3 months at 85% (young: *N* = 11; middle-age: *N* = 10) and the other with full access to food (young: *N* = 8; middle-age: *N* = 10). Three months was chosen to mirror the minimum length of time that rats completing the 5-CSRTT were food restricted, due to the extensive training needed for proper task performance. A summary of the experimental design is displayed in Figure 1. All procedures were conducted in accordance with the National Institute of Health’s (NIH) regulations and were approved by University of Florida’s Institute of Animal Care and Use Committee.

### 5-Choice Serial Reaction Time Task

#### Apparatus

5-CSRTT was conducted in rat operant boxes (Coulbourn Instruments, Whitehall, PA, USA), which had five nose ports on the front panel, grid flooring, a back house light, and a food magazine port in the rear of the box. Each port was equipped with a light to serve as a signal and photobeam sensors to record entrances into the ports. Each box was stored within a sound-attenuating chamber with a fan for aeration and noise dampening. The input and output from each operant box were transmitted to Graphic State 4 software (Coulbourn Instruments, Whitehall, PA, USA) on an Optiplex 9020 computer.

#### Behavioral Paradigm

The 5-CSRTT has been utilized extensively to measure attentional function and impulsivity in rodents, with significant work completed delineating the circuitry and pharmacology of the subcomponents of the task and its measures (Carli et al., 1983; Robbins, 2002). This task has been applied to examine age-related effects on attentional capacity as well (Jones et al., 1995; Muir et al., 1999; Grottick and Higgins, 2002; Guidi et al., 2015b) and thus is a sensitive measure to aging and pharmacological interventions. Rats were habituated to the operant boxes for 15 min, during which time the back house and food magazine lights were illuminated and 10 food pellets (45 mg), serving as reward, were contained within the food magazine. Once rats consumed 10 pellets, they proceeded to the next stage to develop the nose poking response, which entailed nose poking into the food magazine for food pellets on a FR-1 schedule at a rate of approximately three pellets per min for 30 min or until 100 pellets were acquired. After retrieving at least 30 pellets within a session, rats transitioned to the next training stage, which introduced the behavioral contingencies of nose poking to a light stimulus.

All subsequent training stages lasted 30 min (or until 100 trials were completed), and the back house light was illuminated to signal engagement in a trial. Rats initiated a trial with a nose poke into the food magazine, leading to an intertrial interval (ITI) of 5 s prior to illumination of a target hole. Upon nose poking into an illuminated port, the light extinguished, the food magazine was illuminated, and a pellet was delivered. Rats were required to earn 50 rewards for a single behavioral session prior to progressing onto the next training stage. Shaping of the nose poke response included all 5 holes being illuminated at once, with unlimited time to elicit a nose poke. The next training stage required rats to nose poke within 4 min to a single illuminated hole, with an additional minute to respond after the light was extinguished (limited hold period). Omissions and incorrect responses were followed by a time out period, which included all chamber lights extinguishing for 10 s. After rats could nose poke into the illuminated holes within 5 s after it extinguished, the signal duration was shortened and rats were trained to detect signals at 10 s, 2.5 s, and 0.5 s duration. Once rats performed at 50% accuracy on each signal duration individually, they underwent training with variable signal presentations, which included equal presentation of each signal duration across the five ports in a session. Rats performed this variable signal training until the accuracy on all signal durations was >50% with <10% omissions across 5 consecutive training days. Following baseline attentional performance on the variable signal training, rats were injected with LPS (1 mg/kg, i.p.) or saline (1 ml/kg, i.p.) once a week for 4 weeks in total. The weekly injection was delivered 48 h prior to behavioral training for that week to reduce the confounding effect of locomotor-related sickness behavior on attentional performance.

#### Behavioral Analysis

Behavioral performance was averaged across five consecutive training sessions (day 3–7 post-injection). The following behavioral measures were calculated and analyzed: accuracy for each signal type [i.e., (correct/correct+incorrect)*100], percent omissions for each signal type [(omitted trials/omitted+performed trials)*100], percent premature responses [(premature/premature+initiated trials)*100], number of initiated trials, and response and food retrieval latencies. The percent accuracy for each signal type represented sustained attentional capacity across various attentional loads, whereas premature responses indicated impulsivity. To determine if the drug treatment impacted motivation to perform the task, we analyzed the number of trials initiated and completed (i.e., omissions). In addition, latencies were examined to determine changes in motor capacity to perform the task following treatment.

### Chemical Reagent

LPS was acquired from Sigma Aldrich (St. Louis, MO, USA) as a lyophilized powder and solubilized in 0.9% saline (final concentration: 1 mg/ml). It was filtered (0.22 μm) and stored in aliquots at −80°C until shortly before its use, at which point it was stored at 4°C. This LPS originated from *E. coli* serotype O55:B5, with the source strain of CDC 1644–70 and was approximately 500,000 EU/mg.

### Immunohistochemistry

#### Tissue Collection

Forty-eight hours after the final injection (either LPS or saline), rats were anesthetized for tissue collection. This time point was also utilized for the acutely treated (i.e., single injection) rats, which were included to examine the interaction of food restriction on central and peripheral inflammation. Half of the rats were transcardially perfused with 200 ml each of ice-cold 1× phosphate-buffered saline (PBS; pH 7.4) and 4% paraformaldehyde (PFA; pH 7.4). Blood was collected with EDTA-treated syringes *via* the right ventricle prior to perfusion and transferred into plasma tubes (BD, Franklin Lakes, NJ, USA). Plasma tubes were inverted 6–8 times, placed on ice for approximately 30 min, and centrifuged at 1,600 *g* for 15 min at 4°C. Plasma was stored at −80°C until cytokine analysis. The brains were collected, stored in 4% PFA overnight, and then transferred to 30% sucrose. The brains were frozen in OCT compound and stored at −80°C until slicing as 40 μm-thick coronal sections on a cryostat (Microm, Waltham, MA, USA). These sections were stored at −20°C in cryoprotectant solution (15% glucose, 30% ethylene glycol, 0.04% sodium azide, 0.05 M PBS) until later processing using fluorescent immunohistochemistry (IHC) to evaluate the impact of age and peripheral inflammation on astrocytic and microglial activation.

#### Immunofluorescent Procedure

To evaluate the presence of neuroinflammation after peripheral LPS treatment, prefrontal coronal sections were tagged for glial fibrillary acidic protein (GFAP) or ionized calcium binding adaptor molecule (Iba-1) costained with CD68 as astrocyte and microglial markers, respectively. Free-floating sections were rinsed of cryoprotectant solution, incubated in antigen unmasking solution (Vector, Burlingame, CA, USA) at 95°C for 7 min, and rinsed 3 × 5 min in 1× PBS. Sections were blocked in 10% donkey serum, 0.3% Triton, and PBS for 2 h and incubated overnight in primary antibody solution, including 1% bovine serum albumin (BSA), 0.3% triton, and PBS (chicken anti-GFAP: 1:500, ABCam, Cambridge, MA, USA; goat anti-Iba-1: 1:500, ABCam; mouse anti-rat CD68: 1:200, BioRad, Hercules, CA, USA). The sections were rinsed and incubated for 2 h in secondary antibody solution of 1% BSA in PBS (Alexa Fluor 488 donkey anti-chicken, 1:500, Jackson, Bar Harbor, ME, USA; Alexa Fluor 488 donkey anti-goat, 1:500, Life Technologies, Waltham, MA, USA; Alexa Fluor 594 donkey anti-mouse, 1:800, Life Technologies, Waltham, MA, USA). After a final rinse, slices were mounted onto gelatin-coated slides and coverslipped with mounting medium containing DAPI (Vector).

#### Image Analysis

Three representative sections of the medial PFC, specifically the prelimbic cortex (A/P range: +3.7 to +2.0), were processed for each rat (*N* = 4/group for behaviorally performing rats; *N* = 3–6/group for acutely treated rats) for each fluorescent stain. Images were captured at 200× magnification on a Leica DM2500 microscope (Wetzlar, Germany), equipped with a Retiga 4000R camera (QImaging, Surrey, BC, Canada) with QCapture Pro7 software (QImaging, Surrey, BC, Canada). Utilizing NIH *ImageJ*, the size and number of GFAP+ (astrocytes) and Iba-1+ cells (microglia) were measured. Briefly, the image was converted from pixels to micrometer (2.28 pixels/μm for 200× magnification), enhanced to reduce background and improve visualization of the positively-stained cells, and analyzed for count and size. Colocalization of Iba-1 and CD68, which represented [(Iba-1+/CD68+)/Iba-1+ only]*100, was assessed using Adobe Photoshop (San Jose, CA, USA) for 100 Iba-1-positive cells in the prelimbic cortex of each rat.

### Cytokine Assay

In addition to analysis of prefrontal astrocytes and microglia, half of the rats were assessed for shifts in prefrontal cytokine levels. The rats were anesthetized for rapid decapitation as previously conducted (Scheinert et al., 2015), and blood was collected from the carotid artery into plasma tubes. The PFC was collected on ice and frozen immediately in liquid nitrogen for cytokine analysis. Samples were sonicated in a cocktail of phosphatase and proteinase inhibitors, EDTA, 0.1% Tween, and PBS. After sitting on ice for 1 h, samples were centrifuged at 20,000 *g* for 10 min at 4°C, and the supernatant was collected for cytokine analysis.

The Milliplex Multiplex Assay (Millipore, Billerica, MA, USA) was utilized to evaluate age- and treatment-based changes in cytokine levels in blood serum and the PFC. Due to previous studies demonstrating age- and inflammation-induced changes in certain cytokine levels (Deforge and Remick, 1991; Scheinert et al., 2015), the following cytokines and chemokines were included in the panel: eotaxin, IL-1α, IL-1β, IL-6, IL-12p70, IFNγ (interferon gamma), IL-18, MCP-1 (monocyte chemoattractant protein), IP-10, GRO/KC/CINC-1 (growth related oncogene), and RANTES (regulated on activation, normal T cell expressed and secreted; CCL5). Prefrontal cytokine data were converted to pg/μg based on total protein levels acquired from a BSA (Thermo Fisher Scientific, Waltham, MA, USA). Not all cytokines were detected due to the assay’s sensitivity as well as activation levels of certain rats; thus, the final Ns for the analysis varied between cytokines despite initially including 3–4 rats/group for prefrontal homogenates, 4–8/group for the plasma assays. For plasma cytokines, all data were normalized to a z-score for each plate to control for plate variability.

### Statistical Analysis

To investigate the impact of aging and chronic peripheral inflammation on attentional function across the 4 weeks treatment period, a 2 (age) × 2 (treatment) repeated-measures analysis of variance (ANOVA) test was conducted on initiated trials, premature responses, and latencies. In addition, a repeated-measures ANOVA was utilized to examine signal duration-dependent effects for accuracy and omissions, which provided information on performance under high and low attentional load. Changes in peripheral and brain cytokine levels and astrocytic and microglial activation were assessed with a 2 × 2 × 2 ANOVA with age, treatment, and food restriction (*ad libitum* vs. restricted) as between-subject factors. For further analysis, Fisher’s least significant difference *post hoc* test was applied, and a significance cut-off of *p* ≤ 0.05 was used for all statistical tests. The Brown-Forsythe or Welch test was applied for violations of homoscedasticity. For violations of sphericity in the repeated-measures analyses, the Greenhouse-Geisser statistic and degrees of freedom were utilized. All data were analyzed utilizing IBM SPSS statistics 24 software.

## Results

### Effects of Peripheral Inflammation on Sickness Behavior and Attentional Function

Six middle-age rats were removed from the study due to health issues. For those that completed training and testing, no age difference was observed for the number of days to reach criterion. Furthermore, young and middle-age rats performed comparably on accuracy and omissions (*p* > 0.05) prior to treatment.

**Figure 2:**
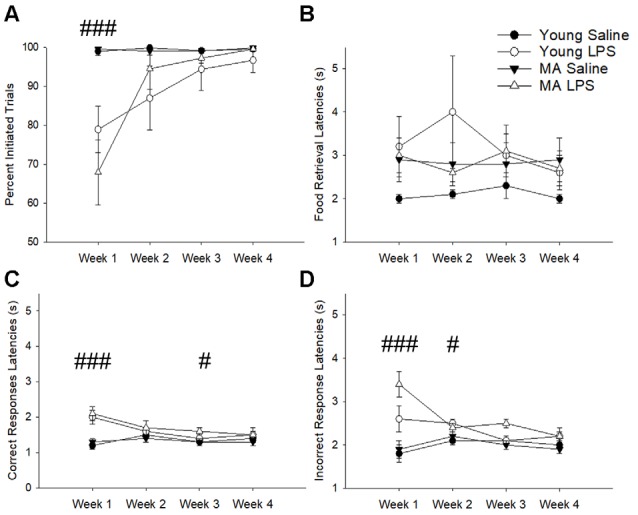
Despite transient sickness behavior, cognitive slowing was characteristic of latter weeks in LPS-treated rats. The first exposure to LPS induced significant sickness behavior effects, marked by a lack of motivation to perform the task as indicated by a reduction in initiated trials **(A)**. To determine if LPS hindered the physical capacity to move, food retrieval latency was analyzed across weeks. This measure did not significantly change between groups **(B)**. Conversely, LPS treatment increased correct response latencies for the first and third weeks **(C)** and incorrect response latencies for the first and second weeks **(D)**, suggesting an overall slowing of cognitive processes without any motor impairment in response to chronic LPS treatment. Data are presented as means ± standard error of the mean (SEM). ^###^*p* < 0.001, ^#^*p* < 0.05 for week by treatment interaction.

The number of trials initiated provided an indication of sickness behavior (i.e., reduced motivation for task performance). A repeated-measures ANOVA found a main effect of treatment (*F*_(1,26)_ = 15.07, *p* = 0.001), a main effect of week (*F*_(1.53,39.81)_ = 11.08, *p* < 0.001), and a week by treatment interaction (*F*_(1.53,39.81)_ = 10.73, *p* = 0.001), in the absence of an age difference. A *post hoc* analysis demonstrated that LPS-treated rats decreased the number of initiated trials during the first week specifically (*p* < 0.001) but recovered in subsequent weeks (*p* > 0.05; Figure 2A). Despite a reduction in motivation to initiate a trial, rats did not display a significant difference in the latency to retrieve a food reward (*p* > 0.05; Figure 2B), which is an indicator of motor function and is another measure for sickness behavior. In addition, no effect of age, treatment, or treatment duration was observed for premature responses, which are sensitive to pharmacological manipulations (Robbins, 2002). Thus, induction of systemic inflammation 48 h prior to task performance reduced motivated behavioral responses only during the first week and no age difference was observed for LPS effects on motivation.

Examination of the latency to nosepoke in response to the cue indicated no effect of age. However, for both correct (Figure 2C) and incorrect response latencies (Figure 2D), there was a main effect of week (correct: *F*_(2.21,57.36)_ = 8.72, *p* < 0.001; incorrect: *F*_(2.01,52.30)_ = 3.46, *p* = 0.04), a main effect of treatment (correct: *F*_(1,26)_ = 12.8, *p* = 0.001; incorrect: *F*_(1,26)_ = 19.37 *p* < 0.001), and an interaction of week by treatment (correct: *F*_(2.21,57.36)_ = 12.36, *p* < 0.001; incorrect: *F*_(2.01,52.30)_ = 7.17, *p* = 0.002). *Post hoc* tests examining treatment effects for each week indicated that compared to saline controls LPS-treated rats were significantly slower in making correct (*p* < 0.001) and incorrect (*p* < 0.001) responses during week 1, likely due to sickness. After sickness-related behavior resolved (i.e., trial initiation normalized), LPS-treated animals still exhibited slower selection of correct stimuli during week 3 (*p* = 0.04) and slower incorrect responses during week 2 (*p* = 0.04). These persistent delays in cue response indicate a potential impact of systemic inflammation on slowing attentional processes.

**Figure 3:**
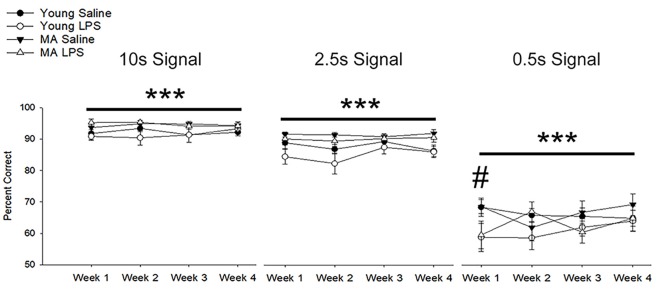
Systemic inflammation impairs attentional accuracy in a signal duration-dependent manner. Young and middle-age rats exhibited a significant signal duration-dependent effect, in which performance accuracy declined as signal duration shortened (*p* < 0.001 for all comparisons). Examination of the impact of systemic inflammation on accuracy demonstrated a significant impairment in LPS-treated rats during the first week of treatment on the shortest signal duration, suggesting that inflammation hinders the capacity to attend during conditions of high attentional load. Data are presented as means ± SEM. ***p < 0.001 for main effect of signal duration, ^#^*p* < 0.05 for treatment interaction.

A repeated-measures ANOVA examined the impact of repeated peripheral inflammation on attentional accuracy across 4 weeks of treatment, with age and treatment as between-subjects factors and weeks and signal duration as within-subjects factors. A significant signal duration-dependent effect was observed (*F*_(1.17,29.04)_ = 477.64, *p* < 0.001), in which accuracy decreased as the signal duration shortened (*p* < 0.001 for all comparisons; Figure 3). A tendency (*p* = 0.07) for an interaction of week by signal duration by injection was observed. *Post hoc* examination of treatment effects for each cue duration indicated that LPS treatment impaired accuracy only for the shortest signal duration. The results suggest that LPS was more likely to impair accuracy when attentional demand was highest.

**Figure 4:**
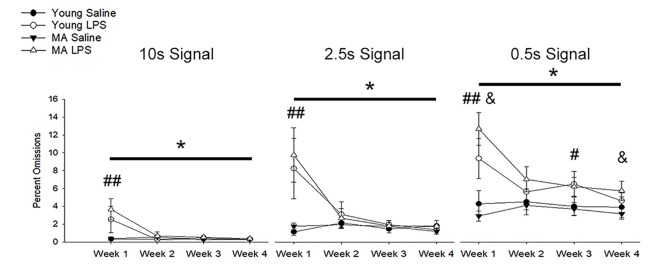
Inflammation increased omission rates, with greater effects for middle-age rats on the shortest signal trial. During the first week of LPS treatment, omission rates significantly increased for all signal trial types (all *p* < 0.01), likely due to sickness behavior inducing a reduction in motivation. In addition, LPS-treated rats displayed greater omissions, which remained high for multiple weeks, on the shortest signal duration of 0.5 s compared to controls (week 1: *p* < 0.001; week 2: *p* = 0.09; week 3: *p* = 0.02; week 4: *p* = 0.08), which may indicate the persistent impairing effect of inflammation on attentional function after sickness behavior resolves. Given their hypothesized enhanced susceptibility to inflammation, middle-age rats were separated from young to evaluate whether they were driving this effect. As expected, while young rats tended to display a sickness-related increase in omissions during week 1 (*p* = 0.08), middle-age rats had higher omission rates for most weeks (week 1: *p* < 0.001; week 2: *p* = 0.07; week 3: *p* = 0.07; week 4: *p* = 0.05). Data are presented as means ± SEM. *p < 0.05, ^#^*p* < 0.05, ^##^*p* < 0.01, interaction of treatment and week; ^&^*p* ≤ 0.05, interaction of age, treatment, and week.

In addition to a reduction in choice accuracy, attentional deficits emerged as a decrease in responding to initiated trials (i.e., omissions). Examination of omission rates indicated a significant main effect of week (*F*_(1.40,36.51)_ = 10.71, *p* = 0.001) and an interaction of week by treatment (*F*_(1.40,36.51)_ = 11.89, *p* < 0.001) due to increased omissions by LPS-treated rats on week 1 (Figure 4). A significant effect of signal duration (*F*_(1.58,40.98)_ = 84.74, *p* < 0.001), a main effect of treatment (*F*_(1,26)_ = 14.94, *p* = 0.001) and an interaction of signal duration and treatment (*F*_(1.58,40.98)_ = 6.57, *p* = 0.006) was observed. LPS-treated rats increased omissions for the shortest signal duration (Figure 4), specifically for weeks 1 (*p* < 0.01) and 3 (*p* = 0.02) and a tendency for increased omissions for week 2 (*p* = 0.09) and week 4 (*p* = 0.08). To determine if LPS induced greater disruption in middle-age rats, as was originally postulated *a priori*, a repeated-measures ANOVA for omissions committed during 0.5 s signal trials was examined within each age group. The results indicated a treatment effect (*F*_(1,14)_ = 18.79, *p* < 0.001) and an interaction of treatment across weeks (*F*_(3,42)_ = 7.93, *p* < 0.001), limited to middle-age animals. The results indicate that middle-age animals were more sensitive to the disruptive effects of LPS for the most cognitively challenging condition.

### LPS Effects on Cytokine Expression

**Figure 5:**
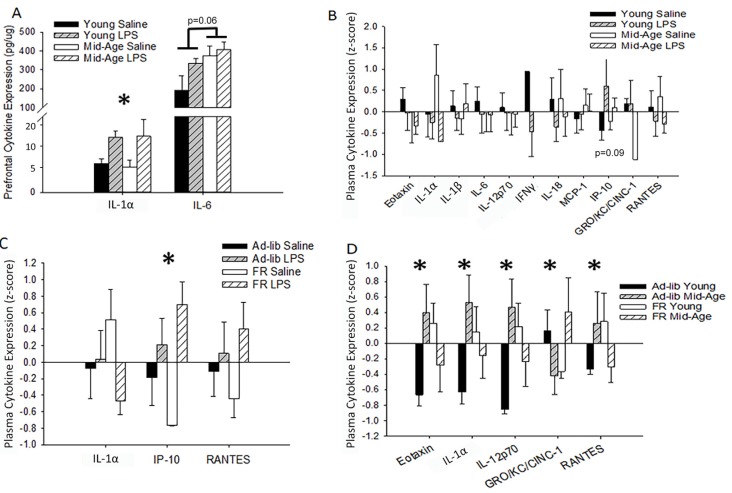
Age, treatment, and food restriction elicit differential effects on brain and plasma cytokine levels. Brain and plasma cytokine levels were assessed for changes in response to systemic inflammation and age. The prefrontal cortex (PFC) of rats chronically treated with LPS exhibited an increase in IL-1α while IL-6 rose specifically in middle-age rats **(A)**. Their plasma, however, was unaffected by both age and treatment **(B)**. Due to the known impact of caloric restriction on inflammation, two cohorts of rats, one food restricted and the other with full access to food, were acutely treated with LPS or saline and compared for altered plasma cytokine levels. Food restriction exacerbated LPS effects for IP-10 **(C)**. However, food restriction mitigated age-specific increases in multiple cytokines, including eotaxin, IL-1α, IL-12p70, and RANTES **(D)**, demonstrating that middle-age rats exhibit heightened levels of pro-inflammatory cytokines and partial caloric restriction attenuates these levels. Data are presented as means ± SEM. *p < 0.05.

To assess changes in cytokine levels with aging and treatment, blood plasma and homogenates from the PFC of repeatedly treated rats were collected 48 h after the final LPS or saline treatment. For the PFC, LPS treatment was associated with increased IL-1α levels (*F*_(1,10)_ = 6.02, *p* = 0.03; saline: 5.31 ± 0.81 pg/μg, LPS: 11.08 ± 2.02), while IL-6 was marginally higher in middle-age rats (*F*_(1,8)_ = 5.05, *p* = 0.06; Figure 5A). Despite shifts in brain cytokines with age and LPS treatment, no significant main effects or interactions of age or treatment on peripheral cytokine levels were observed 48 h after the final injection (Figure 5B). A marginal effect of treatment on plasma IP-10 was noted (*F*_(1,18.6)_ = 3.28, *p* = 0.09), where LPS-treated rats exhibited higher levels.

#### Caloric Restriction Mitigates Plasma Cytokine Expression

LPS-induced changes in peripheral cytokine levels tend to peak at 4–8 h post-injection and resolve after 24 h (Deforge and Remick, 1991; Sharma et al., 1992; Luster et al., 1994; Miller et al., 1997). Thus, the absence of a treatment effect was expected. However, based on previous work from our lab, increases in specific cytokines, such as IL-6, IP-10, and MCP-1, are known to increase in the plasma over the course of aging in Fischer-344 rats (Scheinert et al., 2015). Due to the incorporation of partial food restriction for the behavioral paradigm and the known mitigating effects of caloric restriction on inflammatory responses (Matsuzaki et al., 2001; Horrillo et al., 2011; Willette et al., 2013; Csiszar et al., 2014; MacDonald et al., 2014; Radler et al., 2014; Meydani et al., 2016; Mattson et al., 2018), we investigated the impact of partial food restriction (15% for 3 months) on plasma cytokine levels measured 48 h after one LPS injection. Direct examination of the acutely treated non-performing groups with a 2 (age) × 2 (treatment) × 2 (food restriction; FR) ANOVA confirmed a main effect of treatment (*F*_(1,35.72)_ = 12.59, *p* = 0.001), as well as a marginal interaction of food restriction with treatment (*F*_(1,31)_ = 3.99, *p* = 0.06), for IP-10 levels. Systemic inflammation increased IP-10 levels compared to saline controls, and food restriction exaggerated this effect (*ad-lib* saline vs. FR LPS:* p* = 0.03; FR saline vs. *ad-lib* LPS: *p* = 0.02; FR saline vs. FR LPS: *p* < 0.001; Figure 5C). Age did not impact this measure and thus age was consolidated across groups in Figure 5C.

Predominantly, food restriction interacted with age for a number of plasma cytokines, including eotaxin (*F*_(1,30)_ = 5.75, *p* = 0.02), IL-1α (*F*_(1,30)_ = 4.79, *p* = 0.04), IL-12p70 (*F*_(1,26)_ = 5.75, *p* = 0.02), GRO-KC (*F*_(1,29)_ = 4.44, *p* = 0.04), and RANTES (*F*_(1,31)_ = 4.51, *p* = 0.04; Figure 5D). *Post hoc* analyses demonstrated that food restriction significantly mitigated the extent of the difference in eotaxin, IL-1α, and IL-12p70 levels observed between young and middle-age rats on an *ad-lib* diet (young vs. middle-age *ad-lib*: *p* < 0.03; young vs. middle-age FR: *p* > 0.20). However, the effects were too subtle to detect group differences for RANTES with a *post hoc* test. Interestingly, food restriction increased eotaxin (*p* = 0.05), IL-12p70 (*p* = 0.04), and IL-1α (*p* = 0.09) in young rats compared to middle-age rats, demonstrating a selectively ameliorative effect on an aging inflammatory profile and a stressed response in the young. This age-specific benefit may be due to the fact that the average middle-age laboratory rat is overweight or obese and thus circulate more adipose tissue-derived pro-inflammatory cytokines (Calder et al., 2011; Lumeng et al., 2011; Shin et al., 2015; Starr et al., 2016). Food restriction elicited a distinct shift in GRO-KC plasma levels, whereby food restriction reduced GRO-KC levels in young (*p* = 0.06) but increased it in middle-age rats (*p* = 0.04). Overall, food restriction generally displayed an ameliorative effect on age-related increases in cytokine levels.

### Diverse Shifts in Prefrontal Microglial Activation in Response to Age, Treatment, and Food Restriction

**Figure 6:**
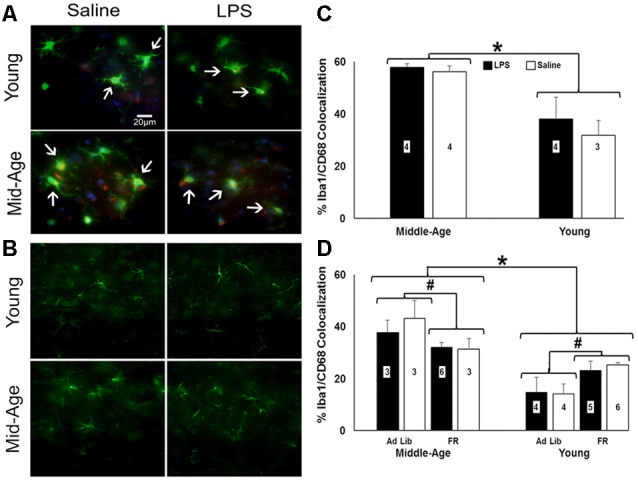
Caloric restriction attenuates PFC microglial activation in middle-age yet instigates it in young. **(A)** Representative images of microglia stained for ionized calcium binding adaptor molecule (Iba-1; green) and CD68-positive (red) and **(B)** astrocytes stained for glial fibrillary acidic protein (GFAP; green) in the PFC of chronically-treated young and middle-age rats. Blue staining indicates DAPI. **(C)** Bar graph of the mean (+ SEM) percent Iba-1+/CD68+ colocalization for rats treated with LPS (filled bars) or saline (open bars). Middle-age rats exhibited heightened colocalization of Iba-1 and the phagocytic marker CD68, suggesting greater microglial activation in the aging PFC. Conversely, examination of food restriction’s impact on acutely treated rats demonstrated a mitigating effect of this age-specific increase **(D)**. Interestingly, young rats displayed the opposite pattern, in which they experienced an increase in Iba-1+/CD68+ colocalization with food restriction. Thus, food restriction may have elicited a potential stress response in young while relieving signs of neuroinflammation in middle-age rats. *Main effect, *p* < 0.05; ^#^interaction of food restriction and age, *p* < 0.05.

Astrocytic (GFAP) and microglial (Iba-1 and CD68) activation markers were examined 48 h after the last injection in repeatedly treated, behaviorally tested rats (Figures 6A,B). No effect of age or treatment was observed for number or size of GFAP+ cells. Similarly, no age or treatment effect was observed for the number of Iba-1+ cells. In contrast, the percentage of microglia displaying phagocytic activity characterized by colocalization of Iba-1 and CD68 shifted by age (*F*_(1,10)_ = 20.43, *p* = 0.001), with middle-age rats exhibiting significantly greater colocalization of Iba-1+ and CD68+ cells relative to young rats (Figure 6C).

To determine if food restriction influenced Iba-1+/CD68+ colocalization, prefrontal slices from acutely treated rats (*N* = 3–6 per group) were examined. A 2 (age) × 2 (treatment) × 2 (food restriction) ANOVA indicated a significant effect of age (*F*_(1,26)_ = 37.84, *p* < 0.001) and an interaction of age and food restriction (*F*_(1,26)_ = 11.70, *p* = 0.002), in the absence of an effect of a single LPS treatment. Again, middle-age rats exhibited increased colocalization; however, food restriction decreased Iba-1+/CD68+ colocalization in middle-age rats (*F*_(1,13)_ = 4.98, *p* = 0.04) and increased colocalization in young rats (*F*_(1,17)_ = 8.95, *p* = 0.008; Figure 6D), suggestive of a heightened stress response akin to the pattern observed with the pro-inflammatory cytokine data (Ganguly et al., 2018).

## Discussion

This experiment examined repeated peripheral inflammation, induced through weekly LPS injections, on peripheral and central inflammatory responses and attentional function between young and middle-age rats. Behavioral measures of the attentional task revealed a temporally distinct manifestation of sickness behavior, limited to the first week of treatment. Reduced motivation for task engagement was noted after the initial LPS treatment, observed as a decrease in the number of trials initiated. Consistent with previous reports, the level of sickness behavior, number of trials initiated and latency to retrieve the reward was not different between young and middle-age animals (Krzyszton et al., 2008; Kinoshita et al., 2009). In subsequent weeks, measures of motivation normalized, consistent with research indicating that tolerance to LPS-induced sickness behavior is similar in young and middle-age animals (Kohman et al., 2010). Sickness likely contributed to impaired accuracy, omissions, and longer decision latencies during the first week. In contrast, impairments in measures of attention persisted beyond week 1. Slower cognitive processing was observed as delayed responses for cue selection, and attentional lapses were observed as omissions on trials with the shortest signal duration, particularly for middle-age animals. Thus, both young and middle-age rats exhibited attentional impairments to repeated systemic inflammation, which were evident days after LPS injections, and impairment was exacerbated with increased cognitive demand and increased age.

Previous studies utilizing old rats have demonstrated a multitude of cognitive deficits with acute peripheral inflammation (Jain et al., 2002; Kohman et al., 2007; Chen et al., 2008; Barrientos et al., 2009a; Tarr et al., 2011); however, few studies have examined middle age, in which behavioral variability begins to emerge (Sparkman et al., 2005; Kohman et al., 2010). LPS produces a robust age-related impairment on the water maze (Sparkman et al., 2005; Chen et al., 2008; D’Avila et al., 2018) and acquisition of an active avoidance task in middle-age animals (Kohman et al., 2007). The relatively mild effect of LPS on attention may be due to the different cognitive processes examined or the use of aversive vs. appetitive motivation. The 5-CSRTT requires food restriction as a motivating factor. Differences in the magnitude of LPS-induced cognitive impairment have been suggested for aversive and appetitive tasks, with milder cognitive deficits (i.e., longer decision latencies) for appetitive tasks (Gahtan and Overmier, 2001).

Importantly, we observed significant mitigating effects of food restriction on inflammatory markers, including microglial activation and inflammatory cytokines. Age differences in plasma cytokines were evident for *ad-lib* rats yet differences disappeared under food restriction. Similarly, examination of Iba-1+/CD68+ microglia of *ad-lib* and food-restricted rats demonstrated that food restriction in middle-age rats reduced microglial activation. In this regard, food restriction can reduce the inflammatory response to LPS (Matsuzaki et al., 2001; Horrillo et al., 2011; Willette et al., 2013; Csiszar et al., 2014; MacDonald et al., 2014; Radler et al., 2014; Meydani et al., 2016; Mattson et al., 2018) and aging (Vasconcelos et al., 2015). However, due to the inherent need for food restriction in the 5-CSRTT, we were unable to examine the potentially neuroprotective effect of food restriction in contrast to an *ad-lib* diet on LPS-induced attentional deficits. Therefore, to understand the full impact of systemic inflammation on attention, tasks that do not need food restriction are required. Regardless, the current study indicates that the impairment of attentional processes outlasts the rise in systemic cytokines, suggesting long-term effects, particularly in middle-age animals.

The mechanism for impaired attention likely involved neuroinflammation and increased production of cytokines. LPS induces an increase in plasma cytokines, which is followed by increased cytokine transcription and expression in the brain (Layé et al., 1994; Gabellec et al., 1995; Goujon et al., 1996; Scheinert et al., 2015). While the rise in systemic cytokines usually resolves within 24 h, the brain response can be long-lasting (Qin et al., 2007; Erickson and Banks, 2011; Fu et al., 2014; Norden et al., 2016). In addition, aging is associated with a chronic rise in brain cytokines (Disabato et al., 2016). Aged animals exhibit an enhanced or prolonged systemic and brain response to an immune challenge (Disabato et al., 2016; Pattabiraman et al., 2017; Barter et al., 2019). Differences in response to chronic neuroinflammation emerge in middle age (Lee et al., 2013; Bardou et al., 2014; Nikodemova et al., 2016). Indeed, expression of genes linked to immune response increase in the hippocampus and PFC during aging, starting in middle age (Blalock et al., 2003; Ianov et al., 2016), suggesting that the aging brain is primed to respond to immune challenges. In the current study, IL-1α and IL-6 increased in the PFC in association with inflammation and aging, respectively. An increase in IL-1α has been reported for chronic neuroinflammation (Bardou et al., 2014). Elevated IL-6 in older animals is consistent with work indicating that IL-6 increases in the aged cortex (Prechel et al., 1996; Ye and Johnson, 1999; Campuzano et al., 2009; Scheinert et al., 2015), heightened levels of IL-6 are associated with cognitive impairments (Singh-Manoux et al., 2014; Puzianowska-Kuźnicka et al., 2016; Lin et al., 2018; Warren et al., 2018), and IL-6 influences prefrontal neuronal activity (Garcia-Oscos et al., 2015; Marsland et al., 2017).

## Conclusion

Systemic inflammation due to weekly LPS injections initially produced behavioral deficits associated with sickness. However, during subsequent weeks, impaired vigilance could be observed as slowed cognitive processing and enhanced omission rates. Both young and middle-age rats exhibited attentional impairments to repeated systemic inflammation, which were evident days after LPS injections, and impairment was exacerbated with increased cognitive demand and increased age. The long-term effect of LPS treatment was associated with increased IL-1α in the PFC, and IL-6 levels were elevated in the PFC in middle-age rats. Food restriction attenuated PFC microglial activation and an age-related increase in plasma cytokines. These results indicate that age-related sensitivity to systemic inflammation influences attentional processes mediated by the PFC. In addition, the results emphasize the need for improved methods for modeling low-level chronic systemic inflammation to effectively examine its impact on attention during aging.

## Data Availability Statement

All data supporting the conclusions of this study are presented in the article.

## Ethics Statement

The animal study was reviewed and approved by Institute of Animal Care and Use Committee at the University of Florida.

## Author Contributions

BY conducted the behavioral training, maintained the injection schedule, sliced and stained the brain tissue, analyzed the cytokine, IHC, and behavioral data, and was a major contributor in writing the manuscript. TF designed the experiment, assisted with data analysis, and was a major contributor in writing the manuscript. All authors read and approved the final manuscript.

## Conflict of Interest

The authors declare that the research was conducted in the absence of any commercial or financial relationships that could be construed as a potential conflict of interest.

## Abbreviations

5-CSRTT: 5-Choice serial reaction time task
ANOVA: Analysis of variance
BSA: Bovine serum albumin
FR: Food restriction
GFAP: Glial fibrillary acidic protein
GRO/KC/CINC-1: Growth-related oncogene
Iba-1: Ionized calcium binding adaptor protein
IHC: Immunohistochemistry
IL: Interleukin
ITI: Intertrial interval
LPS: Lipopolysaccharide
MCP-1: Monocyte chemoattractant protein
NIH: National Institute of Health
PFA: Paraformaldehyde
PBS: Phosphate-buffered saline
PFC: Prefrontal cortex
RANTES: Regulated on activation, normal T cell expressed and secreted.
